# MMT: A Potential Mechanism of Myocardial Fibrosis – Regulation of the TGF-β1/Smad3 Pathway and Prospects for Targeted Therapy

**DOI:** 10.31083/RCM49154

**Published:** 2026-08-05

**Authors:** Ming-rui Zou, Kang Huang, Shuai-shuai Zhao, Bo Wu, Xin Hong, Xue-liang Zhou

**Affiliations:** ^1^Department of Cardiac Surgery, The First Affiliated Hospital, Jiangxi Medical College, Nanchang University, 330209 Nanchang, Jiangxi, China

**Keywords:** TGF-β1/Smad3, macrophage-myofibroblast transition, myocardial fibrosis, therapeutic target

## Abstract

Macrophage-Myofibroblast Transition (MMT) is a process in which macrophages, under the influence of specific factors such as transforming growth factor-beta 1 (TGF-β1) within a fibrotic microenvironment, gradually lose their original immune phenotype and acquire the biological characteristics of myofibroblasts. This transition involves changes in cell morphology, protein expression, and function, representing an important mechanism in the development and progression of tissue fibrosis. The TGF-β1/Smad3 signaling pathway, as a central regulator of fibrosis, plays a key role in MMT by modulating cell proliferation, differentiation, and the synthesis and degradation of extracellular matrix. Recent studies have identified MMT as a newly recognized form of phenotypic switching that contributes significantly to fibrosis in multiple organs, including the heart, lungs, liver, and kidneys. Experimental evidence has confirmed that the TGF-β1/Smad3 pathway directly regulates critical steps in MMT, thereby promoting the progression of fibrosis. However, the specific molecular targets and detailed mechanisms underlying this regulation remain incompletely understood and require further investigation. Elucidating the regulatory network of this pathway may offer new strategies for early intervention and targeted therapy in clinical diseases with fibrosis. However, direct evidence for MMT in human myocardial fibrosis remains limited, and its relative contribution compared to other sources of myofibroblasts requires further elucidation.

## 1. Introduction

Myocardial Fibrosis (MF) is a precursor to heart failure and a common pathological feature of various cardiovascular diseases. It is characterized by excessive collagen deposition and scar formation in the cardiac interstitium, leading to impaired systolic and diastolic function. In recent years, research into the pathological mechanisms and treatment of myocardial fibrosis has become a major focus in cardiovascular research. The molecular mechanisms involved are complex and diverse, involving multiple receptor targets, various cytokines, and numerous signaling pathways [1,2,3]. Currently, Macrophage-Myofibroblast Transition (MMT) is recognized as a crucial cellular phenotypic conversion process involved in organ fibrosis, including cardiac, pulmonary, hepatic, and renal fibrosis. MMT refers to the pathophysiological process where macrophages, under specific environmental conditions, express myofibroblast markers (during the MMT process, macrophages will gradually down-regulate the expression of F4/80, while up-regulating the expression of α-SMA, forming a "F4/80⁺α-SMA⁺" dual-positive transitional state, and eventually transforming into "F4/80⁻/low⁺α-SMA⁺" myofibroblast-like cells), thereby participating in fibrosis [4,5,6]. Although MMT has been observed in experimental models of renal, pulmonary, and hepatic fibrosis, its role in human myocardial fibrosis remains uncertain. Thus, MMT should be regarded as a promising but still evolving concept in cardiac fibrosis research, rather than a definitive proven mechanism.

The TGF-β1/Smad3 signaling pathway is a highly complex, multifunctional core signaling network. Its core biological characteristics include directly regulating target gene expression through precise receptor activation and Smad3 phosphorylation/nuclear translocation, thereby exerting multiple key physiological roles such as inhibiting proliferation, promoting fibrosis, immune regulation, inducing apoptosis, and differentiation. Its functional output is highly dependent on the cell type and microenvironment and forms a complex crosstalk network with numerous other signaling pathways. Dysregulation of this pathway is a core pathological mechanism in several major diseases, particularly pathological fibrosis, making it a highly promising therapeutic target, despite challenges in achieving precise regulation in drug development.

**Fig. 1.**
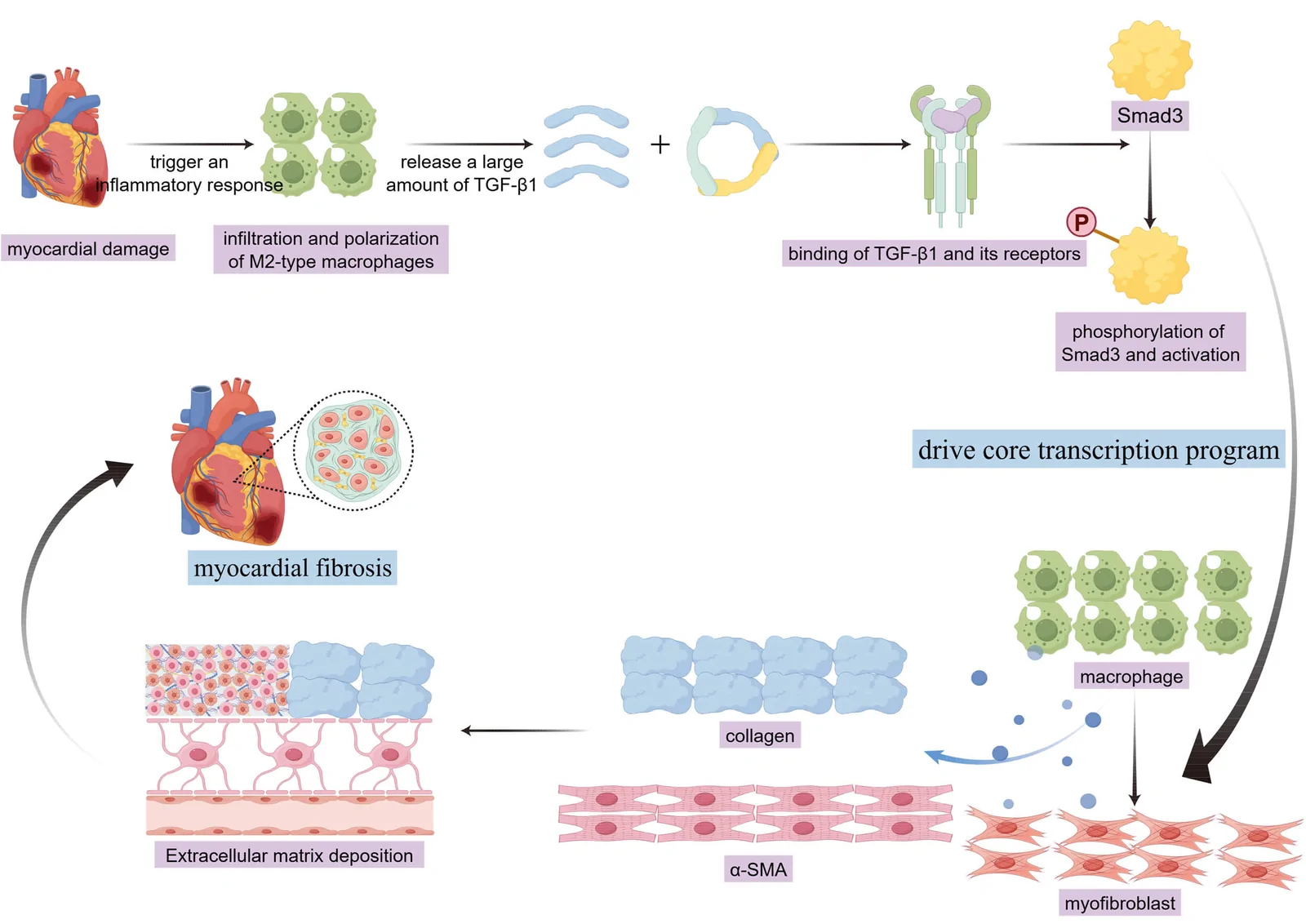
**Schematic diagram of the mechanism of MMT in cardiac fibrosis mediated by the TGF-β1/Smad3 signaling pathway**. MMT, Macrophage-Myofibroblast Transition; TGF-β1/Smad3, Transforming Growth Factor-beta 1/SMAD family member 3. Created by Figdraw.

The TGF-β/Smad pathway plays a crucial role in the MMT process. It can directly mediate the deposition of the extracellular matrix (ECM), autocrine loops, and inflammation-fibrosis coupling by regulating target gene expression, cellular phenotypic switching, and interacting with related pathways. This drives the transition of macrophages into myofibroblasts, establishing it as a core mechanism in fibrotic diseases [7,8]. This review, for the first time, systematically integrates the molecular evidence of the TGF-β1/Smad3 signaling pathway driving myocardial fibrosis through MMT, which is expected to bring about breakthroughs in disease prevention, clinical diagnosis, and future treatments (Fig. 1).

## 2. The Pathological Process of Myocardial Fibrosis

Myocardial fibrosis is a pathological repair process of the heart in response to chronic injury stimuli. It is characterized by excessive deposition, abnormal cross-linking, and disorganized spatial arrangement of the extracellular matrix, ultimately leading to progressive deterioration of cardiac structure and function. At its core lies the overactivation of pro-fibrotic signals (especially the TGF-β/Smad pathway), persistent activation of myofibroblasts, and a severe imbalance in ECM synthesis/degradation/cross-linking [9,10,11]. This process increases myocardial stiffness, causes electrical conduction abnormalities, reduces contractile function, and impairs microcirculation, eventually progressing to heart failure and malignant arrhythmias. It can be broadly divided into three stages:

(1) Initial Injury Stage: Under specific conditions or pathogenic factors (such as ischemia/hypoxia, inflammatory mediators, or mechanical stress), cardiomyocytes undergo apoptosis or necrosis. The injured site recruits M1 macrophages and neutrophils, which release various inflammatory factors (such as TNF-α, IL-6, and proteases), further exacerbating myocardial tissue damage.

(2) Fibrosis Activation Stage: Cardiac fibroblasts (CFs) differentiate into myofibroblasts (expressing α-Smooth Muscle Actin (α-SMA)+) under the stimulation of factors such as Transforming Growth Factor-β (TGF-β), Angiotensin II (Ang II), and Platelet-Derived Growth Factor (PDGF), acquiring contractile and ECM secretory capabilities. A decrease in elastic collagen and inhibition of matrix metalloproteinase expression lead to excessive ECM deposition.

(3) Fibrotic Remodeling Stage: Collagen diffusely deposits in the myocardial interstitium, and necrotic areas are replaced by scar tissue (reparative fibrosis), affecting myocardial compliance (increased stiffness) and ultimately leading to impaired cardiac systolic and diastolic function.

Recent studies have found that macrophages can directly transform into ECM-secreting cells via MMT, which becomes a new driver of fibrotic remodeling (detailed in Section 3.1).

## 3. The Process of Macrophage-Myofibroblast Transition

### 3.1 Macrophage Polarization and Activation

As a key component of the immune system, macrophages are involved in fibroblast activation, ECM remodeling, and the resolution of fibrosis [12,13]. They are essential for maintaining cardiovascular homeostasis and triggering tissue damage. Macrophages are highly heterogeneous and constitute a crucial part of the innate immune system. During the initiation and progression of fibrosis, activated macrophages are a significant source of chemokines and other inflammatory mediators that drive the initial cellular response after injury. In addition, macrophages participate in interactions with other cells, primarily by stimulating the proliferation and activation of fibroblasts, promoting their differentiation into collagen-producing myofibroblasts [14,15]. During the resolution phase of fibrosis, anti-fibrotic macrophage subsets degrade ECM components, inhibit ECM-producing cells, and clear apoptotic cells to prevent or reverse fibrosis. In hypertensive mouse model hearts, circulating monocyte-derived macrophages express myofibroblast markers (α-SMA, Transgelin). Lineage tracing experiments have confirmed that hypertension promotes the transition of macrophages into myofibroblasts. Macrophage-specific knockout of ALKBH5 (an m6A demethylase) identified circulating monocyte-derived macrophages as the primary source of pathological transition [16,17]. m6A modification may also amplify the pro-fibrotic effects of TGF-β1 by regulating inflammatory factors such as IL-6. IL-6 can enhance TGF-β1-induced Smad3 phosphorylation, forming a positive feedback loop that exacerbates MMT and ECM deposition [18,19]. This mechanism manifests as excessive collagen accumulation and decreases cardiac function in models of myocardial fibrosis. The traditional M1/M2 dichotomy is insufficient to describe macrophage diversity in fibrosis. Single-cell technologies reveal that macrophages can be divided into pro-fibrotic and anti-fibrotic subpopulations, influencing the fibrotic process by regulating fibroblast activation, ECM remodeling, and inflammatory responses. Both pro-inflammatory (M1) and anti-inflammatory (M2) macrophages possess transformative potential, though some studies confirm that M2 macrophages are the primary precursors involved in MMT [20,21]. Although single-cell data support the existence of MMT, lineage tracing evidence remains limited, and both supporting and skeptical viewpoints need to be balanced and validated in future studies.

### 3.2 Co-expression of Phenotypic Markers and Cytoskeletal Reorganization

Induced by specific factors (such as cytokines and growth factors), some macrophages lose their original phenotype, change from round to spindle-shaped or stellate, and express a series of characteristic proteins, transforming into cells with pro-fibrotic functions. These proteins include: α-Smooth Muscle Actin (α-SMA), which integrates into stress fibers, conferring strong contractile force; Vimentin, a marker of mesenchymal cells; Fibroblast Specific Protein 1 (FSP1), involved in the cell transformation process; and increased synthesis of procollagen [22,23,24]. Accompanying this transition, the cells massively secrete collagen and fibronectin, leading to excessive deposition of the extracellular matrix (ECM), increased tissue stiffness, and subsequent structural damage, functional loss, and tissue fibrosis.

### 3.3 Acquisition of Functional Myofibroblast Characteristics

Activated myofibroblasts are the primary ECM-producing cells that participate in fibroblast differentiation. Upon transitioning into macrophage-derived myofibroblasts, cells undergo significant functional and morphological changes, becoming core effector cells driving tissue fibrosis [5,25]. Their morphology shifts from round or amoeboid to spindle-shaped or stellate, with enhanced migratory capacity aiding infiltration into injured sites. The cells acquire strong contractile properties, evidenced by the formation of α-SMA stress fibers and assembly of focal adhesion structures, enabling them to exert traction forces on the ECM via integrin-mediated connections, leading to tissue contracture. Concurrently, the cells’ ability to synthesize and secrete ECM components (such as collagen and fibronectin) is substantially increased, directly contributing to tissue stiffness and structural disruption. The cells then switch to secreting pro-fibrotic factors like TGF-β and downregulate some classical pro-inflammatory cytokines, shifting their overall phenotype from pro-inflammatory towards maintaining and advancing the fibrotic process. MMT is a recently identified form of cellular phenotypic conversion in fibrotic disease research. It refers to the process by which macrophages acquire the biological characteristics of myofibroblasts within specific inflammatory or injury microenvironments [26,27,28]. The main process involves: (1) Tissue injury triggering an inflammatory response; (2) Recruitment of macrophages to the injury site as part of the inflammatory response; (3) Macrophages, under the influence of cytokines and growth factors such as TGF-β, acquire myofibroblast characteristics, such as expressing α-smooth muscle actin and other myofibroblast markers. Our current understanding is that macrophages (originating from bone marrow hematopoietic stem cells, belonging to the hematopoietic lineage) and myofibroblasts (originating from local tissue mesenchymal stem cells, epithelial cells, or endothelial cells, belonging to the mesenchymal lineage) are cells of different differentiation lineages. The direct, large-scale conversion of one terminally differentiated cell type into another completely different lineage (transdifferentiation) is biologically rare and lacks a clear, widely accepted direct evidence in both normal physiology and pathology. In some fibrotic lesions (such as myocardial, pulmonary, and renal fibrosis), activated macrophages (particularly M2 type) and myofibroblasts may both express some common cell markers (e.g., both may express α-SMA at certain stages). This process reveals a direct link between immune cells and fibrosis, providing a new perspective for understanding fibrotic mechanisms.

### 3.4 Evidence and Correlates of MMT in Human Cardiac Pathologies

While the majority of mechanistic evidence for MMT originates from genetically engineered mouse models, a critical question remains regarding its existence and relevance in human myocardial fibrosis. Direct lineage-tracing experiments, performed in animals, are not feasible in human patients. Therefore, the evidence in human hearts is necessarily correlative and relies on the identification of cells exhibiting a hybrid or transitional phenotype, consistent with the MMT process observed in experimental studies.

Immunohistochemical Evidence from Human Tissue: Studies on endomyocardial biopsies or explanted hearts from patients with various etiologies of heart failure (e.g., ischemic cardiomyopathy, hypertensive heart disease, dilated cardiomyopathy) have provided suggestive morphological data. Several reports have documented the co-localization of canonical macrophage/monocyte markers (such as CD68 or CD163) with the myofibroblast marker α-smooth muscle actin (α-SMA) within fibrotic interstitial regions [29,30,31]. These “double-positive” cells are often found at the border zones of fibrosis or within active scar tissue. It is crucial to interpret these findings with caution: co-staining represents a phenotypic correlation, not definitive proof of lineage conversion, as it could theoretically result from very close apposition of two distinct cell types, phagocytosis of debris, or non-specific staining. Nevertheless, their presence aligns spatially and phenotypically with the MMT hypothesis derived from animal models.

Transcriptomic Evidence from Single-Cell RNA Sequencing (scRNA-seq): The advent of scRNA-seq has provided a more powerful tool to interrogate cellular identities and disease states in human heart failure without relying solely on a limited set of protein markers. Re-analyses of public scRNA-seq datasets from human failing hearts have revealed subpopulations of myeloid-derived cells that co-express transcripts associated with both inflammatory functions (e.g., CD14, *AIF1/IBA1*, CD163) and extracellular matrix remodeling (e.g., COL1A1, COL3A1, POSTN, TNC) [30,32]. These clusters exhibit a gene signature that is distinct from classical M1/M2 macrophages and resting fibroblasts, potentially representing a transcriptomic intermediate state akin to cells undergoing MMT. The identification of such hybrid populations in human samples significantly strengthens the biological role of MMT in human cardiac pathology.

Clinical Correlation and Future Directions: The clinical significance of these putative MMT-related cells is an area of active investigation. Preliminary correlative studies suggest that the abundance of macrophages with a “fibrotic” transcriptomic signature or the density of CD68+/α-SMA+ cells may be associated with the severity of fibrosis quantified by cardiac magnetic resonance (CMR) imaging, the degree of left ventricular dysfunction, or adverse clinical outcomes. Future, prospectively designed studies that integrate high-dimensional cellular phenotyping (e.g., CITE-seq, spatial transcriptomics) with precise imaging-based fibrosis metrics and longitudinal patient follow-up are essential to establish whether MMT-related cells are merely bystanders or active drivers of disease progression in humans. Until such direct functional evidence is available, the human data should be interpreted as strongly supportive of the clinical relevance of the MMT concept, justifying further translational research, while acknowledging that it remains a mechanistic hypothesis yet to be definitively proven in human hearts.

## 4. Biological Characteristics of the TGF-β1/Smad3 Signaling Pathway

The core of the TGF-β1/Smad3 signaling pathway lies in TGF-β1 (ligand) activating its receptor, which then primarily phosphorylates Smad3 (downstream signal transducer). The phosphorylated Smad3 forms a complex with Smad4, translocates into the nucleus, and regulates the transcription of target genes. It widely participates in regulating various biological processes, including cell proliferation, differentiation, apoptosis, migration, adhesion, and ECM synthesis [33,34].

Several core pathways promote fibrosis, such as the TGF-β/Smad pathway (upregulating α-SMA and collagen genes), the Wnt/β-catenin pathway (promoting fibrotic gene transcription via β-catenin nuclear translocation), and inflammatory pathways (NLRP3 inflammasome activation leading to IL-1β secretion, stimulating fibroblast activity), alongside others such as histone modification and miRNA regulation. The TGF-β/Smad pathway acts as a core driver of fibrosis and also plays a key role in the biology of MMT. This article focuses on the biological role of the TGF-β1/Smad3 signaling pathway in the MMT process [35,36,37].

### 4.1 Core Components and Signal Transduction Process

• Ligand: TGF-β1 is a representative member of the TGF-β superfamily. It is a pleiotropic cytokine usually secreted in a latent form and requires activation (e.g., by integrins, proteases, or acidic environments) to bind receptors.

• Receptors:

Type II Receptor (TβRII): Possesses constitutive kinase activity. TGF-β1 first binds to TβRII. Type I Receptor (TβRI / ALK5): Recruited by TβRII and binds the ligand, forming a heterotetrameric complex. TβRII phosphorylates and activates the kinase domain of TβRI.

• Smad Signal Proteins: Smad2 and Smad3 are the primary R-Smads transducing TGF-β1 signals. TβRI directly phosphorylates the C-terminal SSXS motif of Smad3 (phosphorylation of Smad2 sometimes requires co-receptors like SARA). This phosphorylation is a key step for downstream signal propagation.

• The phosphorylated Smad3 (p-Smad3) binds to the common mediator Smad4, forming a heterotrimeric complex (p-Smad3/Smad3/Smad4), which is translocated into the nucleus with the help of importins.

• Transcriptional Regulation: Inside the nucleus, the p-Smad3/Smad4 complex needs to cooperate with DNA-binding co-factors (such as Forkhead box O (FoxO), AP-1 family members, Runt-related transcription factor (RUNX), etc.) to effectively bind to Smad Binding Elements (SBEs-the DNA sequence recognized by Smad transcription factors) on the promoter/enhancer regions of target genes.

• Recruitment of Transcriptional Regulatory Complexes: The p-Smad3/Smad4 complex itself has weak transcriptional activation capacity. It primarily serves as a platform to recruit transcriptional co-activators or co-repressors, ultimately determining whether target genes are activated or repressed through mechanisms like histone acetylation/deacetylation [38,39,40,41,42,43,44].

### 4.2 Core Biological Functions

#### 4.2.1 Pro-fibrotic

The TGF-β1/Smad3 pathway is a key molecular engine driving tissue fibrosis. It strongly promotes the transition of macrophages into myofibroblasts, specifically manifested by:

(1) ECM Synthesis: Upregulating the expression of various ECM protein genes, such as Collagen I, III, IV (COL1A1, COL3A1, etc.), Fibronectin (FN1), and Laminin [45,46,47].

(2) Inhibition of ECM Degradation: Inducing the expression of Plasminogen Activator Inhibitor-1 (PAI-1), inhibiting plasminogen activators (like uPA/tPA), thereby reducing the production and activation of plasmin and Matrix Metalloproteinases (MMPs). Simultaneously, it induces the expression of Tissue Inhibitors of Metalloproteinases (TIMPs), collectively promoting ECM accumulation [48,49,50].

(3) Activation and Differentiation of Myofibroblasts: Stimulating the differentiation of fibroblasts and pericytes into secretory myofibroblasts, which are the primary source of excessive ECM deposition in fibrotic tissues [51,52].

#### 4.2.2 Inhibition of Cell Proliferation

This is one of the most classic biological effects of TGF-β1, particularly in epithelial cells, endothelial cells, hematopoietic cells, and immune cells. It primarily works by inducing the expression of Cyclin-Dependent Kinase Inhibitors (CDKIs) such as p15^INK4B^, p21^CIP1/WAF1^, and p27^KIP1^, leading to cell cycle arrest in the G1 phase [53,54].

#### 4.2.3 Immune Regulation

• Anti-inflammatory/Immunosuppressive: Inhibits the proliferation and activation of T cells and B cells; promotes the differentiation and function of regulatory T cells (Tregs); suppresses the activity of dendritic cells, macrophages, and NK cells. This is important for maintaining peripheral immune tolerance and suppressing autoimmune reactions [55].

• Pro-inflammatory Effects: Under certain conditions (e.g., early tissue injury), TGF-β1 can also promote the expression of pro-inflammatory factors and the recruitment of macrophages/neutrophils [56].

#### 4.2.4 Induction of Apoptosis

Can induce apoptosis in certain cell types (e.g., hepatocytes, T cells, B cells), which is crucial for embryonic development, maintaining tissue homeostasis, and immune tolerance.

Regulation of Cell Differentiation: Participates in the differentiation processes of various cell types, such as inducing Epithelial-Mesenchymal Transition (EMT, important in development and cancer metastasis), osteoblast differentiation, and chondrocyte differentiation [57,58].

Maintenance of Tissue Homeostasis: Plays a central role in tissue repair after injury and maintains normal structure by balancing cell proliferation, apoptosis, and ECM synthesis/degradation. However, when the signal is excessive or persistently activated, it can lead to pathological states (e.g., fibrosis, cancer) [59,60].

Epithelial-Mesenchymal Transition: The TGF-β1/Smad3 pathway is one of the key factors inducing EMT, achieved by downregulating epithelial markers (e.g., E-cadherin), upregulating mesenchymal markers (e.g., N-cadherin, Vimentin, Fibronectin), and EMT-related transcription factors [61,62].

### 4.3 Association With Pathological Diseases

#### 4.3.1 Fibrotic Diseases

Overactivated or sustained activation of the TGF-β1/Smad3 signal is a core driver of fibrosis in nearly all organs (liver, kidney, lung, heart, skin). Its ability to promote ECM synthesis and inhibit degradation directly leads to organ dysfunction and scar tissue formation. In organs like the kidney, lung, and liver, the TGF-β1/Smad3 pathway can induce epithelial cells to undergo EMT, enabling them to acquire a mesenchymal cell phenotype and participate in fibrosis formation [42,63].

#### 4.3.2 Cardiovascular Diseases

In hypertension and atherosclerosis, TGF-β1 influences the phenotypic switching of vascular smooth muscle cells (VSMCs) (from contractile to synthetic), proliferation, and migration, leading to vascular wall thickening and stiffening. It plays a dual role in atherosclerosis: on one hand, it may have a protective effect by inhibiting inflammation and stabilizing plaques; on the other hand, it may promote fibrous cap formation, leading to stenosis and intimal hyperplasia. Furthermore, under pressure overload, TGF-β1 signaling participates in the development of pathological cardiac hypertrophy [15,64,65].

#### 4.3.3 Autoimmune and Inflammatory Diseases

TGF-β1 is a core cytokine for maintaining immune tolerance and homeostasis. Deficiencies in its signaling can lead to immune system dysregulation. TGF-β1 is essential for the development and function of Treg cells, which are crucial for suppressing autoimmune responses. Smad3 signaling is necessary for the suppressive function of Tregs. Insufficient or dysregulated TGF-β1 signaling fails to effectively inhibit the differentiation and function of pro-inflammatory T cells, leading to massive production of inflammatory cytokines (e.g., IL-6, IL-17, IFN-γ) and affecting B cell class switching and antibody production [66].

In diseases such as multiple sclerosis, rheumatoid arthritis, and inflammatory bowel disease, dysregulation of this pathway (often manifesting as excessive immune suppression or ineffective suppression) contributes to pathological changes. Its anti-inflammatory function also makes it a potential therapeutic target [67].

#### 4.3.4 Cancer

The TGF-β1/Smad3 pathway plays a classic “double-edged sword” role in cancer, with its effects being distinctly stage-dependent.

Early Stage - Tumor Suppressive Role: Acts via Smad3-dependent pathways to induce the expression of CDKIs (e.g., p15, p21), inhibit the expression of cyclins (e.g., Cyclin D1), thereby arresting the cell cycle in G1 phase. It also promotes apoptosis and differentiation [68].

Late Stage - Tumor Promoting Role: When cancer cells evolve, they can hijack the TGF-β1 signal to promote malignant progression. Through Smad3 and other pathways (e.g., PI3K/AKT), cancer cells acquire migratory and invasive capabilities, key steps in metastasis. TGF-β1 is one of the strongest immunosuppressive factors, inhibiting the activity of cytotoxic T cells and NK cells, promoting the differentiation of Tregs, and helping tumor cells evade immune attack. It also upregulates Vascular Endothelial Growth Factor (VEGF) to nourish tumor growth and recruits and alters stromal cells like Cancer-Associated Fibroblasts (CAFs), forming a microenvironment that supports tumor growth and metastasis [69,70].

The TGF-β1/Smad3 signaling pathway is an extremely complex cellular communication system, the delicate balance of which is crucial for health. Its dysregulation—whether overactivation or functional deficiency—leads to a range of serious pathological conditions from fibrosis and cancer to autoimmune diseases. Understanding the pathophysiological characteristics of this pathway is essential for developing safe and effective targeted therapies and represents a current hotspot and challenge in translational research.

### 4.4 Interactive Network of TGF-β1/Smad3 With Fibrosis-Related Pathways

#### 4.4.1 TGF-β1/Smad3 and MAPK/ERK Pathway

TGF-β1 can not only signal through the canonical Smad pathway but also directly activate Mitogen-Activated Protein Kinase (MAPK) family pathways such as Extracellular signal-Regulated Kinase (ERK), c-Jun N-terminal Kinase (JNK), and p38. Among these, activated p38 MAPK can phosphorylate the linker region of Smad proteins. This modification serves as a key cross-talk mechanism, significantly enhancing Smad activity and its ability to drive gene transcription. Concurrently, the MAPK pathway itself can independently regulate the expression of certain pro-fibrotic genes by phosphorylating other transcription factors. Thus, TGF-β1 promotes fibroblast proliferation, survival, and differentiation through this precise network of Smad-MAPK synergy and complementation, which significantly influences the fibrotic process [71,72,73].

#### 4.4.2 TGF-β1/Smad3 and PI_3_K/Akt Pathway

TGF-β1, by activating the PI_3_K/Akt signaling pathway, strongly inhibits the apoptosis of myofibroblasts via Akt phosphorylation, allowing their long-term survival. Additionally, activated Akt can indirectly regulate EMT through non-Smad pathways. This inhibition of apoptosis is a key mechanism ensuring the persistent survival of myofibroblasts and their excessive secretion of ECM, thereby maintaining and promoting the progression of fibrosis [74,75].

#### 4.4.3 TGF-β1/Smad3 and Wnt/β-catenin Pathway

β-catenin mediates Smad3 nuclear translocation: After Wnt ligand activation, β-catenin accumulates and enters the nucleus, binding to T-cell Factor (TCF)/Lymphoid Enhancer-binding Factor (LEF) transcription factors, directly enhancing SMAD3 gene transcription. The β-catenin/Smad3 complex binds to the promoter regions of collagen genes (COL1A1, COL3A1), amplifying ECM synthesis [76].

In myocardial fibrosis, Wnt1 overexpression increases β-catenin levels by 2.8-fold, concurrently increasing p-Smad3 nuclear localization and accelerating fibroblast transformation [77].

#### 4.4.4 TGF-β1/Smad3 and JAK/STAT Pathway

Certain pro-inflammatory cytokines (e.g., IL-6, IL-13) participate in the fibrotic process by activating the JAK/STAT signaling pathway, and TGF-β1 can further upregulate the expression of STAT proteins. Among them, STAT3 can functionally synergize with the TGF-β1 downstream protein Smad3, effectively mediating the transition from inflammation to fibrosis, jointly promoting myofibroblast activation and excessive ECM deposition [78].

#### 4.4.5 TGF-β1/Smad3 and Notch Pathway

TGF-β1 increases the proliferation, invasion, adhesion capacity, and Collagen I secretion of cardiac fibroblasts (CFs). These effects are inhibited by overexpression of the Notch Intracellular Domain (NICD). Furthermore, NICD knockdown enhances endogenous Smad3 phosphorylation in CFs, while TGF-β1-induced Smad3 phosphorylation is antagonized by NICD overexpression. Conversely, activation of endogenous NICD in CFs is antagonized by Smad3, and TGF-β1-induced NICD inactivation is antagonized by Smad3 knockdown. Co-immunoprecipitation shows interaction between NICD and Smad3, and immunostaining shows nuclear co-localization of NICD and Smad3 in CFs. Functional antagonism between NICD and Smad3 on CF phenotype has also been demonstrated [78,79].

#### 4.4.6 TGF-β1/Smad3 and the Renin-Angiotensin-Aldosterone System (RAAS)

Ang II directly activates TβRII: Ang II phosphorylates TβRII via the AT1R receptor, independently of the TGF-β ligand. The Mineralocorticoid Receptor (MR), upon activation, upregulates TGF-β1 expression, forming an “Ang II → MR → TGF-β1 → Smad3” positive feedback loop; i.e., aldosterone induces Smad3 phosphorylation [80].

**Fig. 2.**
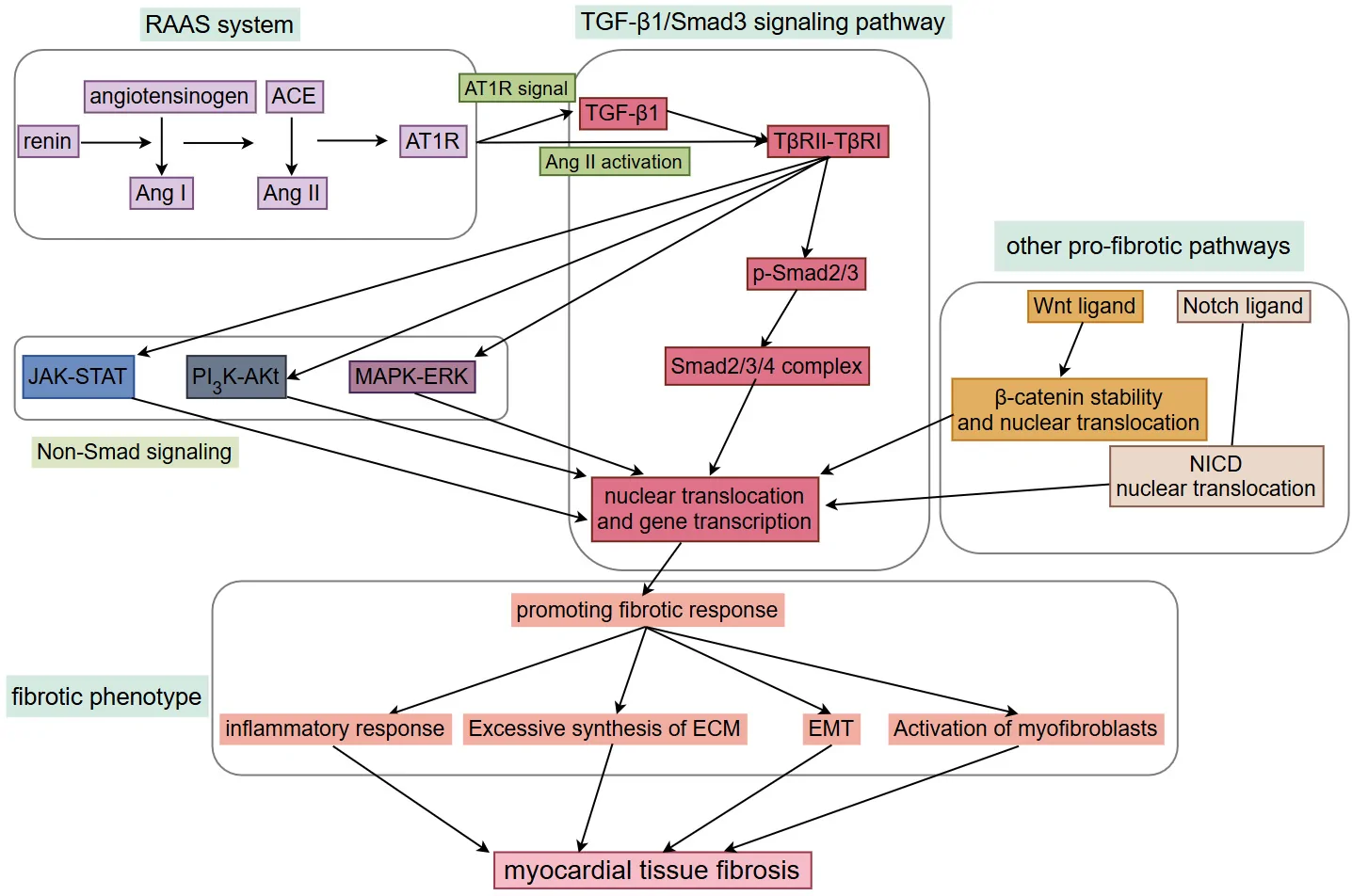
**Schematic diagram of the interactive network of TGF-β1/Smad3 and other fibrosis-related pathways**. Created by Figdraw.

In hypertensive models, Losartan (an AT1R antagonist) reduces p-Smad3 levels by up to 65%, significantly inhibiting the transition of macrophages into α-SMA+ cells [81] (Fig. 2).

#### 4.4.7 TGF-β1/Smad3 and m6A Modification Pathway

A close bidirectional positive feedback relationship exists between the TGF-β1/Smad3 signaling pathway and m6A RNA modification, with defined molecular coordinates and a sequential timeline [82,83].

Positive regulation (TGF-β→m6A): Activated TGF-β1/Smad3 signaling directly transactivates the expression of the core m6A methyltransferase complex, notably METTL3. The phosphorylated Smad3/Smad4 complex binds to specific Smad Binding Elements (SBEs) within the METTL3 gene promoter, driving its transcription. This leads to an overall increase in cellular m6A deposition, particularly on mRNAs harboring the consensus RRACH (R=G/A; H=A/U/C) motif.

Reverse amplification (m6A→TGF-β): The installed m6A marks are recognized by “reader” proteins such as YTHDF1/2 or IGF2BP1. Critically, the mRNAs encoding key components of the TGF-β1 pathway itself (e.g., TGFB1, TGFBR1, SMAD2/3) contain highly conserved RRACH motifs in their 3’untranslated regions (3’UTRs). m6A modification at these sites enhances the stability and translation efficiency of these transcripts via reader protein binding. Conversely, m6A modification can also promote the decay of mRNAs encoding inhibitory molecules, such as SMAD7, a negative feedback regulator of the pathway. This post-transcriptional regulation creates a powerful self-reinforcing loop [84].

**Fig. 3.**
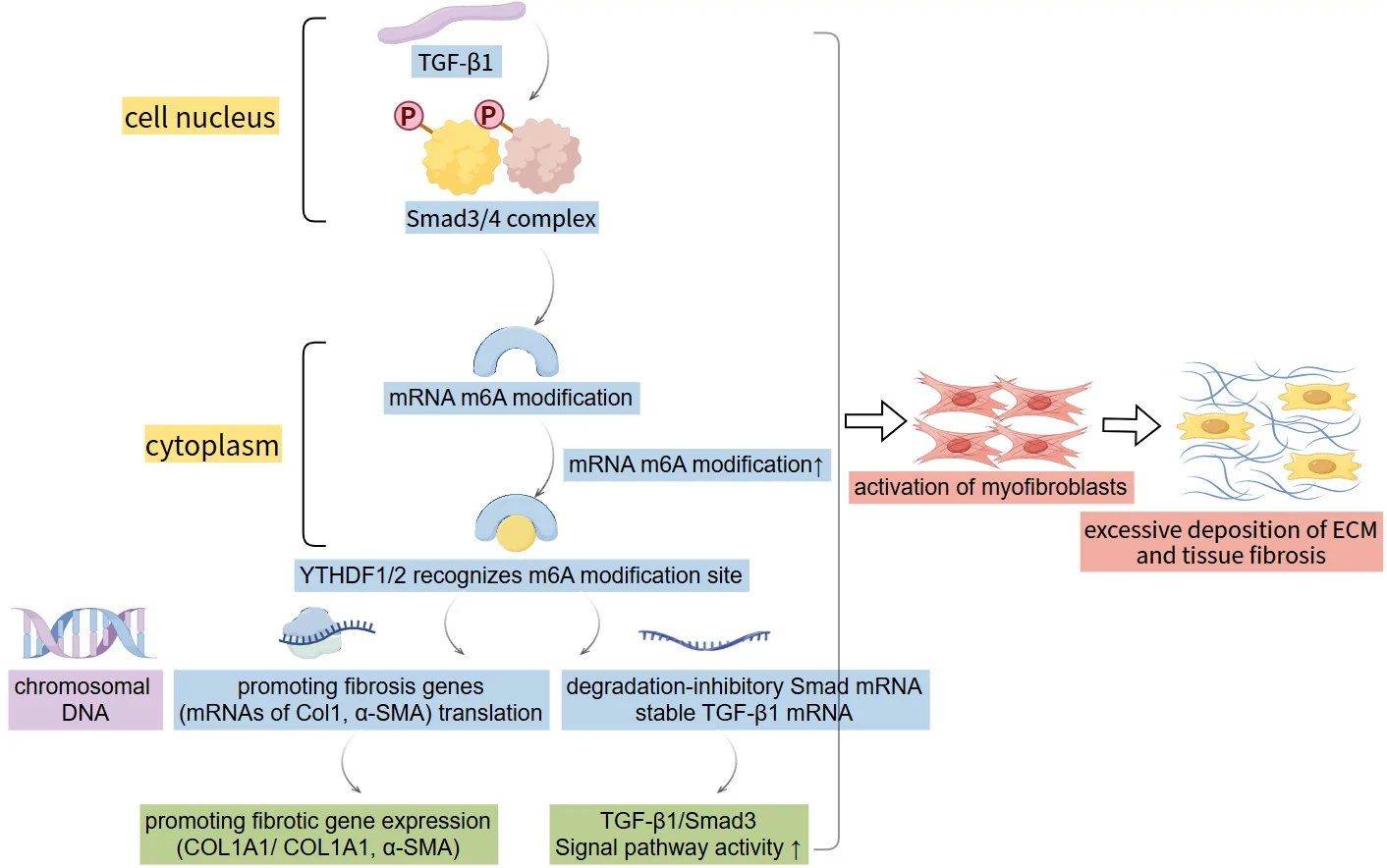
**Schematic diagram illustrating the interactive mechanism between the TGF-β1/Smad3 signaling pathway and the m6A modification pathway**. Created by Figdraw.

Regulate the timeline: In the early phase of fibrotic stimulation, rapid Smad3 activation may induce a preliminary wave of METTL3 expression. The resulting increase in m6A then stabilizes and amplifies the signal output of the TGF-β1 pathway components, locking the cell into a sustained pro-fibrotic transcriptional program. This sequential amplification is a key mechanism driving the progression and persistence of organ fibrosis (Fig. 3).

#### 4.4.8 TGF-β1/Smad3 and Epigenetics (O-GlcNAc Glycosylation)

O-GlcNAc modification acts as a dynamic, reversible “metabolic sensor” that fine-tunes the activity of the TGF-β1/Smad3 pathway through direct steric competition at the post-translational level [85,86,87,88].

Molecular competitive sites: O-GlcNAc transferase (OGT) catalyzes the addition of a single N-acetylglucosamine moiety to serine or threonine residues of target proteins. On Smad3, O-GlcNAcylation occurs predominantly within its linker region, a domain rich in Ser/Thr-Pro sites that are also targets for proline-directed kinases (e.g., CDKs, MAPKs), and is in close proximity to the C-terminal SSXS motif phosphorylated by TβRI. O-GlcNAcylation of specific linker region residues (e.g., T179) can physically hinder the access of TβRI to the C-terminal motif or alter Smad3’s conformational stability, thereby directly inhibiting its phosphorylation and subsequent nuclear translocation.

Energy sensing and regulation logic: The donor substrate for O-GlcNAcylation, UDP-GlcNAc, is synthesized via the hexosamine biosynthesis pathway (HBP), which integrates inputs from glucose, amino acid, fatty acid, and nucleotide metabolism. Therefore, the level of Smad3 O-GlcNAcylation directly reflects cellular nutrient status. Under acute metabolic stress (e.g., myocardial ischemia), an adaptive increase in O-GlcNAcylation may serve as a protective brake to prevent excessive TGF-β1/Smad3-driven apoptosis or fibrosis. Conversely, in sustained pathological metabolic states (e.g., diabetic cardiomyopathy), chronically elevated O-GlcNAcylation may dysregulate this fine-tuning mechanism and contribute to maladaptive signaling.

**Fig. 4.**
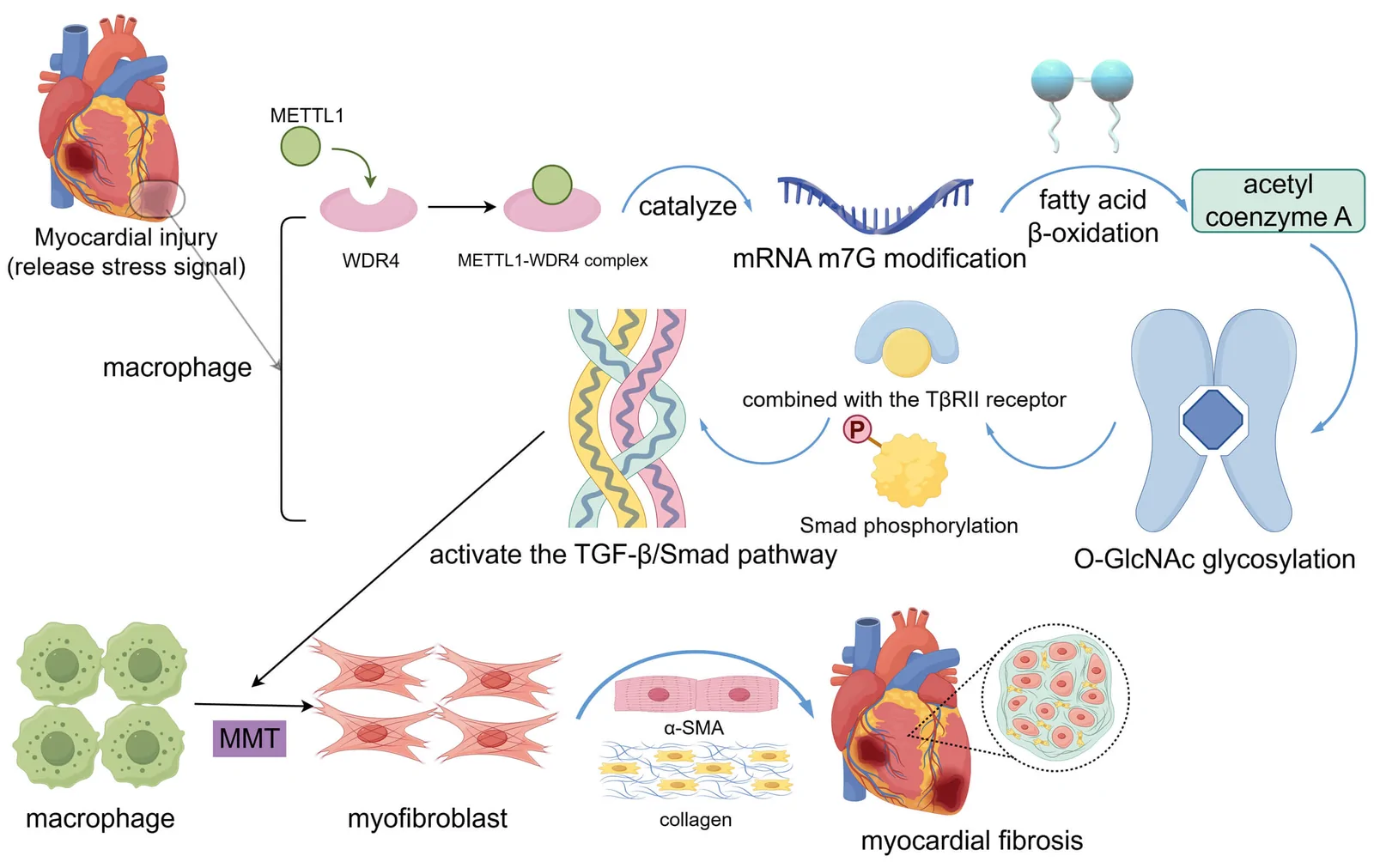
**Schematic diagram illustrating the mechanism by which METTL1-mediated m7G modification regulates O-GlcNAcylation and affects the myocardial fibrosis MMT process**. Created by Figdraw.

Bidirectional interaction: The TGF-β1/Smad3 pathway can, in turn, regulate the expression of OGT and O-GlcNAcase (OGA), the enzyme that removes the modification, forming a complex feedback loop. This crosstalk tightly couples cellular decisions to the metabolic microenvironment (Fig. 4).

### 4.5 TGF‑β1/Smad3‑Mediated Transcriptional Reprogramming in Macrophages During MMT

While the canonical TGF-β1/Smad3 pathway is operational in many cell types, its ability to drive the specific phenotypic conversion of macrophages into myofibroblasts hinges on a unique transcriptional reprogramming event. This process extends beyond simple Smad3 phosphorylation and nuclear translocation; it requires the coordinated interaction of phospho-Smad3 (p-Smad3) with cell-type-specific transcription factors and epigenetic regulators within the macrophage nucleus, ultimately redirecting gene expression from an immune profile to a pro-fibrotic one.

Cooperative Transcription Factors Define Cellular Specificity: Smad3 itself possesses weak, low-specificity DNA-binding affinity. Its transcriptional outcome is largely determined by cooperative partnerships with lineage-defining or lineage-appropriate transcription factors. In macrophages undergoing MMT, p-Smad3/Smad4 complexes are postulated to engage with key macrophage lineage factors, effectively “repurposing” them to silence macrophage identity genes and activate myofibroblast programs.

Interactions with Macrophage Lineage Factors: Core macrophage transcription factors such as PU.1 (encoded by SPI1) and members of the CCAAT/enhancer-binding protein (C/EBP) family (e.g., C/EBPβ) are critical for establishing and maintaining macrophage identity. During MMT, TGF-β1 signaling may lead to functional crosstalk or direct complex formation between p-Smad3 and these factors. This interaction could result in the suppression of classic macrophage markers (e.g., CD68, *ADGRE1/F4/80*) while simultaneously enabling the recruitment of this transcriptional machinery to the enhancers/promoters of myofibroblast signature genes [2].

Synergy with Profibrotic Transcription Factors: p-Smad3 synergizes with other transcription factors that are either upregulated or activated during fibrosis. A prime example is RUNX1. Evidence indicates that RUNX1 is essential for promoting MMT in fibrotic models, and it can physically interact with Smad3 to co-occupy regulatory elements of genes encoding α-SMA (ACTA2) and collagen I (COL1A1), thereby directly driving their expression. Similarly, components of the AP-1 complex (e.g., c-Fos/c-Jun) frequently cooperate with Smad3 to regulate a broad spectrum of ECM-related and inflammatory genes, serving as a nexus for integrating TGF-β signals with other pathways.

Context of Ligand Activation: The source and mode of TGF-β1 activation are likely crucial in initiating MMT within the macrophage niche. Unlike fibroblasts, macrophages residing in the fibrotic myocardium are exposed to TGF-β1 that is predominantly activated locally from latent complexes. This activation can be mediated by integrins (e.g., αvβ8 expressed on adjacent cells or macrophages themselves), proteases, or the acidic microenvironment of an injury. This spatially restricted signal may provide a potent, context-specific trigger for the MMT program in recruited macrophages, distinguishing it from the responses of resident fibroblasts.

Epigenetic Reinforcement: The transcriptional switch is stabilized by accompanying epigenetic changes. The p-Smad3 complex can recruit co-activators such as p300/CBP, which catalyze histone acetylation, opening chromatin at profibrotic gene loci. Concurrently, it may facilitate the repression of macrophage-specific genes through mechanisms involving histone deacetylases (HDACs). This epigenetic layer ensures the relative stability of the acquired myofibroblast phenotype [89].

Conclusion of this subsection: In summary, the TGF-β1/Smad3 pathway does not act in isolation to induce MMT. Instead, it functions as a master transcriptional coordinator within macrophages, leveraging pre-existing lineage-determining factors and synergizing with induced pro-fibrotic factors to execute a precise genetic switch. This collaborative model explains how a ubiquitous signaling pathway can elicit the cell-type-specific outcome of macrophage-to-myofibroblast transition. Future research elucidating the exact composition and genomic binding sites of these Smad3-containing transcriptional complexes in macrophages will be key to identifying highly specific targets for disrupting MMT.

## 5. MMT: A Potential Intervention Target?

Tissue fibrosis is a common terminal pathological manifestation of various chronic diseases, characterized by excessive ECM deposition and the destruction of organs. The traditional view holds that myofibroblasts primarily originate from the activation of local fibroblasts or via Epithelial-Mesenchymal Transition (EMT) [90]. However, recent studies have found that macrophages, under specific conditions, can express the biological characteristics of myofibroblasts and participate in the disease’s pathological process, i.e., the MMT phenomenon. This discovery provides a new perspective on fibrotic mechanisms and may become a new target for anti-fibrotic therapy. For example, fibrosis could potentially be improved by targeting the regulation of the macrophage transition process or by early clearance of specific macrophage subpopulations (pro-fibrotic groups). Macrophage-specific Smad3 silencing can effectively hinder MMT and prevent disease progression [74,91].

Other studies suggest that macrophages can exert anti-fibrotic effects through the targeted recruitment of specific subpopulations. However, fibrotic mechanisms are complex, and the dynamic phenotypes and functional heterogeneity of macrophages pose challenges for targeted therapy (e.g., off-target risks), necessitating a balance between anti-fibrotic effects and tissue repair. Furthermore, differences exist in α-SMA and collagen protein expression between *in vivo* and *in vitro* experiments. Future work requires an in-depth analysis of the regulatory mechanisms of transition and the pathological functions of the transitioned cells.

Research on the role of MMT in the pathogenesis of fibrotic diseases has mainly focused on the upstream and downstream signaling pathways activated by the TGF-β/Smad signal. Although macrophages play important physiological functions, TGF-β1 remains a crucial cytokine. Given the central role of the TGF-β1/Smad3 pathway in major diseases like fibrosis and tumor metastasis, inhibiting this pathway has become an important strategy in drug development. This includes small molecule kinase inhibitors, monoclonal antibodies, soluble receptors, targeting other nodes of the Smad pathway (e.g., molecules targeting Smad3 phosphorylation, complex formation, nuclear translocation), and targeting downstream effector molecules. Specific approaches could involve using CRISPR-Cas9 technology to validate the role of key MMT genes (like ALKBH5) in myocardial models or integrating AI models to predict crosstalk between the TGF-β1/Smad3 pathway and the Renin-Angiotensin-Aldosterone System (RAAS) [92] (see Table 1, Ref. [93,94,95,96,97,98,99,100]).

**Table 1.** **Potential intervention strategies and mechanistic implications [93,94,95,96,97,98,99,100]**.

Intervention strategy	Target/Mechanism	Experimental evidence	Primary challenges and considerations	Clinical translation & updates
m6A Modification Inhibitors	ALKBH5 inhibitor (targeting IL-11).	Reduces fibrosis degree in hypertensive models.	Target Specificity: ALKBH5 has broad substrate specificity; Tissue Delivery: Requires macrophage/lesion-specific delivery systems.	Promising preclinical progress: ALKBH5 small‑molecule inhibitors (e.g., STM2457) have shown safety in early‑phase hematological malignancy trials (NCT05584111). In fibrosis models, ALKBH5 knockdown/inhibition attenuates cardiac, renal, and pulmonary fibrosis, yet no fibrotic‑indication clinical trials are ongoing.
Antibody Therapy	TGF-β1 neutralizing antibody.	Effective in animal models, significant toxicity in clinical trials.	Significant Safety Concerns: Systemic inhibition leads to dose-limiting toxicities, such as skin hyperplasia, vasculopathy, and increased risk of autoimmunity activation.	Clinical example: Fresolimumab (GC1008) showed antifibrotic potential in early‑phase IPF and FSGS trials, but further development was halted due to systemic toxicity. Current strategies shift toward local delivery or bispecific antibody designs.
TβRI Kinase Inhibitors	Competitively inhibits TβRI kinase activity, preventing phosphorylation of Smad2/3, thus blocking downstream signaling.	In preclinical cancer models, inhibits tumor growth/metastasis, reverses EMT, enhances chemo/immunotherapy efficacy. Also effective in fibrosis models.	Lack of Target Selectivity: May affect other kinases; Escape Mechanisms: Cancer cells or myofibroblasts may escape via ligand upregulation or alternative pathway (e.g., MAPK) activation.	Clinical progress: Galunisertib (LY2157299) showed some efficacy in Phase II HCC trials and is explored in pancreatic cancer/glioblastoma combinations. Its application in fibrosis (e.g., cardiac) remains at the preclinical‑to‑early translational stage.
Targeting Downstream Effectors (e.g., Integrin Inhibitors)	Blocks key molecules mediating TGF-β activation. Many cytokines are secreted latent, requiring integrins (e.g., αvβ6, αvβ8) for cell-surface activation.	αvβ6/β8 integrin knockout or inhibitors effective in lung/liver fibrosis models, with better safety profile than direct TGF-β inhibition, as it primarily blocks local activation in pathological contexts, not systemic signaling.	Tissue Specificity: Requires identification of the dominant integrin in the diseased organ; High Development Hurdle: Design and optimization of high-affinity inhibitors.	Clinical advance: αvβ6 integrin inhibitor BG00011 (BRAVOS) has been evaluated in a Phase II IPF trial (NCT03573505), showing preliminary antifibrotic activity and favorable safety. Such agents are considered safer due to their localized mechanism of action.
Targeting MMT-specific nodes	Disrupting the unique transcriptional program driving MMT within macrophages (e.g., knocking down RUNX1, inhibiting ALKBH5).	Macrophage-specific Smad3 knockout can block MMT; RUNX1 has been demonstrated to play a key role in MMT.	Cell-Specific Delivery: Requires efficient delivery of therapeutics to specific macrophage subsets within the lesion; Physiological Role of Target: Long-term impact on immune homeostasis needs evaluation.	Medium–High translational potential: Represents the most specific direction but heavily depends on breakthroughs in cell‑targeting technologies. No clinical‑stage drugs directly targeting MMT yet, but MMT‑associated markers (e.g., RUNX1, ALKBH5) are emerging as novel biomarker/therapeutic targets validated by single‑cell studies.
Agonist/Function Restoration Strategy (for autoimmune diseases)	Aims to enhance TGF-β signaling to restore immune tolerance. E.g., using low-dose TGF-β, receptor agonists, or stabilizing Smad signaling.	In autoimmune animal models (e.g., EAE, mimicking MS), administering TGF-β1 or therapies promoting its production can alleviate disease.	Carcinogenesis Risk: Long-term enhancement of TGF-β signaling may theoretically promote fibrosis or tumor progression; Limitations in Administration Routes.	Limited clinical exploration: Due to carcinogenic/profibrotic risks, direct TGF‑β or receptor agonist development is rare. Current approaches focus on Treg adoptive transfer or indirect tolerance induction rather than direct pathway enhancement.

Abbreviations: ALKBH5, Alpha-ketoglutarate-dependent dioxygenase alkB homolog 5; RUNX1, Runt-related transcription factor 1; αvβ6\αvβ8, Integrin alpha V beta 6/Integrin alpha V beta 8; EAE, Experimental Autoimmune Encephalomyelitis; MS, Multiple Sclerosis; EMT, Epithelial-Mesenchymal Transition.

Another challenge lies in balancing efficacy and toxicity, as TGF-β has irreplaceable roles in maintaining physiological homeostasis. Current research hotspots include defining the conversion threshold for pro-fibrotic/anti-fibrotic macrophage subpopulations, achieving selective inhibition in specific tissues or of specific downstream effects, and developing context-dependent drugs.

The central role of TGF-β1/Smad3 signaling in MMT makes it a prime therapeutic target for mitigating fibrosis. However, translating this concept into safe and effective therapies faces a fundamental challenge: the pleiotropic and context-dependent functions of TGF-β1. Beyond driving fibrosis, TGF-β1 is indispensable for immune homeostasis, surveillance against autoimmunity, wound healing, and vascular integrity. Consequently, systemic inhibition of the TGF-β pathway has been associated with severe, dose-limiting toxicities in preclinical and clinical studies, including the emergence of autoimmune-like syndromes, impaired wound repair, and vascular abnormalities (e.g., bleeding, telangiectasia). Therefore, the primary hurdle is no longer proving the pathway’s involvement, but achieving a therapeutic window where pathological signaling in fibrotic niches is inhibited without disrupting its vital physiological roles. This necessitates a strategic shift from broad pathway blockade towards more nuanced approaches, such as: (1) targeting the local activation of TGF-β1 within fibrotic tissues; (2) disrupting MMT-specific downstream transcriptional complexes; or (3) exploiting the unique phenotypic state of macrophages undergoing transition.

While most mechanistic strategies targeting MMT or the TGF-β1/Smad3 axis remain in preclinical exploration, the clinical translation landscape for fibrotic diseases is dynamically evolving. Notably, drug development in oncology and pulmonary fibrosis provides critical precedents and pipelines for cardiac applications. Table 1 has been updated to include the most advanced clinical evidence, highlighting both the promising leads and the substantial gaps that remain for myocardial fibrosis-specific therapies. Table 1 summarizes various possible strategies and clearly assesses their mechanism principles, associated challenges, and the latest clinical trial data.

The updated clinical landscape underscores a clear divergence: broad TGF-β pathway inhibition faces insurmountable safety barriers, while context-dependent strategies like integrin inhibition are forging a viable path forward. The progress of αvβ6/8 integrin inhibitors in IPF represents the most tangible clinical precedent for an anti-fibrotic therapy rooted in modulating TGF-β activation. For newer strategies like m6A modulation or MMT-specific targeting, the path is longer. Their translation will likely depend on two key enablers: (1) borrowing safety and compound optimization insights from parallel oncology programs; (2) breakthroughs in delivery technologies (e.g., lipid nanoparticles, cell-specific ligands) to achieve the necessary cellular and tissue specificity. Future clinical trials in myocardial fibrosis should prioritize these targeted, mechanistically precise approaches over broad pathway blockade.

## 6. Controversies and Unresolved Issues Regarding MMT

Macrophage-Myofibroblast Transition (MMT), as an emerging concept in fibrotic disease research, has garnered attention in recent years, but it still faces numerous controversies and unresolved questions.

### 6.1 Direct Evidence and Controversy

MMT, as an emerging concept in fibrotic disease research, has received increased attention in recent years, but it still faces numerous controversies and unresolved questions, chief among them being its quantitative contribution to the overall myofibroblast pool. Estimates of bone marrow-derived (and thus potentially MMT-originated) myofibroblasts in fibrotic tissues vary dramatically across studies, ranging from less than 10% to over 50% of the total α-SMA+ cells. This wide disparity is less likely to reflect fundamental biological differences between models and more likely attributable to critical methodological variations in detection and lineage tracing.

Limitations of Marker Co-localization: The initial evidence for MMT often relies on immunohistochemical co-staining for a macrophage marker (e.g., CD68, F4/80) and a myofibroblast marker (α-SMA). While suggestive, this approach has inherent pitfalls. First, α-SMA is not exclusively specific to myofibroblasts; it is also expressed in vascular smooth muscle cells, pericytes, and a subset of activated mesenchymal stem cells. Second, CD68 can be expressed at low levels by certain non-myeloid cells under stress. Third, and most importantly, co-localization at the light microscopy level cannot definitively distinguish a single bi-phenotypic cell from two tightly apposed distinct cells, or from a macrophage that has phagocytosed cellular debris containing α-SMA. Therefore, marker studies can overestimate the frequency of MMT and are considered necessary but insufficient proof [32,101].

Divergence in Lineage Tracing Models: Genetic lineage tracing is the gold standard for establishing cellular origins, yet differences in experimental design directly impact the conclusions that can be drawn.

Promoter Specificity: Commonly used Cre drivers target different segments of the myeloid lineage. Cx3cr1-CreER primarily labels monocytes and a subset of tissue-resident macrophages; Lyz2-Cre (also known as LysM-Cre) has a broader expression, marking granulocytes and most macrophages; while *F4/80-Cre* targets mature tissue macrophages. The size and nature of the labeled “source pool” differ significantly, affecting the potential yield of traced myofibroblasts [102].

Recombination Efficiency and Fidelity: No Cre line is 100% efficient or perfectly specific. Inefficient recombination can lead to false-negative results (underestimation), while “leaky” expression in non-target cell types (e.g., due to transient developmental expression or epigenetic silencing) can cause false-positive lineage assignment (overestimation).

Persistence of the Lineage Tracer: The choice of the reporter is crucial. If a long-lived fluorescent protein (e.g., tdTomato) is used, a cell that fully transdifferentiates into a myofibroblast and completely silences its macrophage gene program will still be labeled. This can lead to an overestimation of the current contribution of MMT-derived cells to the active myofibroblast population, as it captures all historical conversions regardless of their present state.

Model and Organ-Specific Context: The contribution of MMT may genuinely vary across different organs (kidney vs. heart vs. lung), types of injury (pressure overload vs. metabolic vs. ischemic), and stages of disease (acute vs. chronic). A mechanism that is predominant in one context may be minor in another [1,4].

In conclusion, the current controversy surrounding the quantitative importance of MMT is largely rooted in these methodological complexities. The reported spectrum of contributions likely represents the combined effect of different technical approaches rather than a single biological truth. Moving forward, convergent evidence from multiple, rigorously controlled and complementary lineage tracing strategies—coupled with single-cell omics to define transitional states—will be essential to obtain a reliable estimate and to determine whether MMT is a major driver or a subsidiary pathway in myocardial fibrosis.

### 6.2 Regulatory Mechanisms and Molecular Pathways of MMT

Core Pathway: The TGF-β1/Smad3 signaling pathway is widely regarded as the key molecular engine regulating MMT. The duration and intensity of TGF-β1 exposure might be critical factors determining whether macrophages ultimately polarize towards an M2 phenotype or undergo MMT [103,104].

Interaction with Other Pathways: MMT is not regulated by a single pathway in isolation. Studies show that other signaling pathways like JAK-STAT, and epigenetic regulations such as m6A RNA methylation (e.g., ALKBH5-mediated IL-11 mRNA demethylation) are also involved. How these pathways form “cross-talk” with the TGF-β1/Smad3 pathway to precisely regulate MMT is a current research hotspot and challenge.

Initial Triggers: Which initial factors or microenvironmental signals (e.g., specific cytokine combinations, mechanical stimuli, metabolites) most effectively initiate the MMT process requires further exploration.

### 6.3 Pathological Function and Heterogeneity of MMT

Functional Duality: The traditional M1/M2 dichotomy might be overly simplistic. Macrophages undergoing MMT may exhibit a unique hybrid phenotype or functional state, simultaneously possessing certain immunoregulatory properties and strong fibrogenic capacity [105]. Whether these MMT-derived myofibroblasts differ functionally, in persistence, and reversibility from myofibroblasts of other origins is currently poorly understood.

Cellular Heterogeneity: Single-cell sequencing technologies reveal high heterogeneity and plasticity in macrophages [106]. Which macrophage subpopulations (e.g., M2 type, especially M2a subtype-Anti-inflammatory/fibrotic macrophage subtypes) are more prone to undergo MMT? Do specific precursor cells exist that are inclined towards MMT? These questions await in-depth analysis.

### 6.4 Technical Challenges and Research Models

Technical Limitations: Current research heavily relies on marker co-expression (e.g., CD68/α-SMA) to identify MMT cells. However, this method might be prone to misjudgment, such as from cell fusion or signals from very closely apposed cells. Although lineage tracing is a more reliable technique, its specificity, sensitivity, and accuracy still require continuous optimization.

Limitations of Model Systems: Most MMT evidence comes from mouse models. The relevance and importance of these findings in human diseases require more validation in human tissue samples. Simultaneously, existing *in vitro* cell culture models might not fully replicate the complex *in vivo* fibrotic microenvironment.

### 6.5 Therapeutic Potential of Targeting MMT

Target Specificity and Safety: Key pathways like TGF-β also play important roles in physiological homeostasis. How to precisely target the MMT process while avoiding disruption of its normal physiological functions is a major challenge in drug development. For instance, direct broad inhibition of TGF-β signaling might cause significant off-target effects and toxicity [107,108].

Therapeutic Time Window: Fibrosis is a dynamic process. What is the optimal therapeutic window for intervening in MMT? Is it during the initiation phase, progression phase, or during attempts to promote its reversal? This requires a deeper understanding.

## 7. Summary and Outlook

Myocardial fibrosis can be caused by various pathological injuries and is present in almost all types of cardiovascular diseases. Its pathological process relies on the synergistic interaction between macrophages and myofibroblasts, which collectively drive myocardial remodeling and fibrosis. The traditional view is that macrophages indirectly regulate fibroblast activity via paracrine signaling (e.g., growth factors, cytokines) to promote collagen deposition or directly transform into fibroblast-like cells. However, the transformed cells might be pathogenic fibroblasts, and the lack of fine genetic regulation leads to collagen deposition and adverse myocardial remodeling. Numerous studies have now confirmed that, under specific conditions, a subset of macrophages loses their original phenotype and function, acquiring myofibroblast biological characteristics (such as expressing α-SMA, secreting ECM), thereby participating in collagen production and driving pathological fibrosis [109].

Based on the academic controversies and unresolved issues surrounding MMT, precisely targeting key molecular pathways (such as components of the TGF-β and RAAS axes, matrix metalloproteinases, and inflammatory mediators) has become an important therapeutic strategy for reversing or delaying myocardial fibrosis and improving cardiac remodeling. Myocardial fibrosis is a common final pathway for many cardiovascular diseases. Its core mechanisms involve TGF-β1/Smad3 signaling activation, RAAS system dysregulation, inflammation-fibrosis conversion, and ECM metabolic imbalance. Current treatments primarily aim to slow progression. Targeted intervention against TGF-β1, the RAAS system, and ECM regulatory molecules may represent future areas of breakthrough research for this disease.
